# Robotic, transanal, and laparoscopic total mesorectal excision for locally advanced mid/low rectal cancer: European multicentre, propensity score-matched study

**DOI:** 10.1093/bjsopen/zrae044

**Published:** 2024-05-28

**Authors:** Nicola de’Angelis, Francesco Marchegiani, Aleix Martínez-Pérez, Alberto Biondi, Salvatore Pucciarelli, Carlo Alberto Schena, Gianluca Pellino, Miquel Kraft, Annabel S van Lieshout, Luca Morelli, Alain Valverde, Renato Micelli Lupinacci, Segundo A Gómez-Abril, Roberto Persiani, Jurriaan B Tuynman, Eloy Espin-Basany, Frederic Ris, European MRI and Rectal Cancer Surgery (EuMaRCS) Study Group, Giorgio Bianchi, Eva Martí-Martínez, Teresa Torres-Sánchez, Juan Carlos Sebastián-Tomás, Isacco Maretto, Gaya Spolverato, Simone Guadagni, Alejandro Solis

**Affiliations:** Unit of Robotic and Minimally Invasive Digestive Surgery, Department of Surgery, Ferrara University Hospital, Ferrara (Cona), Italy; Unit of Colorectal and Digestive Surgery, DIGEST Department, Beaujon University Hospital (AP-HP), Clichy, France; University Paris Cité, Paris, France; Unit of Colorectal Surgery, Department of General and Digestive Surgery, Hospital Universitario Doctor Peset, Valencia, Spain; Biosanitary Research Institute, Valencian International University (VIU), Valencia, Spain; General Surgery Unit, Fondazione Policlinico Universitario Agostino Gemelli IRCCS, Rome, Italy; General Surgery 3, Department of Surgery, Oncology and Gastroenterology, University of Padua, Padua, Italy; Unit of Robotic and Minimally Invasive Digestive Surgery, Department of Surgery, Ferrara University Hospital, Ferrara (Cona), Italy; Unit of Colorectal Surgery, Department of General and Digestive Surgery, University Hospital Vall d’Hebron, Universitat Autonoma de Barcelona, Barcelona, Spain; Unit of Colorectal Surgery, Department of General and Digestive Surgery, University Hospital Vall d’Hebron, Universitat Autonoma de Barcelona, Barcelona, Spain; Department of Surgery, Amsterdam University Medical Center, Location Vrije Universiteit Amsterdam, Amsterdam, The Netherlands; General Surgery Unit, Department of Translational Research and New Technologies in Medicine and Surgery, University of Pisa, Pisa, Italy; Department of Digestive Surgery, Groupe Hospitalier Diaconesses, Croix Saint-Simon, Paris, France; Department of Digestive Surgery, Groupe Hospitalier Diaconesses, Croix Saint-Simon, Paris, France; Unit of Colorectal Surgery, Department of General and Digestive Surgery, Hospital Universitario Doctor Peset, Valencia, Spain; General Surgery Unit, Fondazione Policlinico Universitario Agostino Gemelli IRCCS, Rome, Italy; Department of Surgery, Amsterdam University Medical Center, Location Vrije Universiteit Amsterdam, Amsterdam, The Netherlands; Unit of Colorectal Surgery, Department of General and Digestive Surgery, University Hospital Vall d’Hebron, Universitat Autonoma de Barcelona, Barcelona, Spain; Service of Abdominal Surgery, Geneva University Hospitals and Medical School, Geneva, Switzerland

## Abstract

**Background:**

Total mesorectal excision (TME) is the standard surgery for low/mid locally advanced rectal cancer. The aim of this study was to compare three minimally invasive surgical approaches for TME with primary anastomosis (laparoscopic TME, robotic TME, and transanal TME).

**Methods:**

Records of patients undergoing laparoscopic TME, robotic TME, or transanal TME between 2013 and 2022 according to standardized techniques in expert centres contributing to the European MRI and Rectal Cancer Surgery III (EuMaRCS-III) database were analysed. Propensity score matching was applied to compare the three groups with respect to the complication rate (primary outcome), conversion rate, postoperative recovery, and survival.

**Results:**

A total of 468 patients (mean(s.d.) age of 64.1(11) years) were included; 190 (40.6%) patients underwent laparoscopic TME, 141 (30.1%) patients underwent robotic TME, and 137 (29.3%) patients underwent transanal TME. Comparative analyses after propensity score matching demonstrated a higher rate of postoperative complications for laparoscopic TME compared with both robotic TME (OR 1.80, 95% c.i. 1.11–2.91) and transanal TME (OR 2.87, 95% c.i. 1.72–4.80). Robotic TME was associated with a lower rate of grade A anastomotic leakage (2%) compared with both laparoscopic TME (8.8%) and transanal TME (8.1%) (*P* = 0.031). Robotic TME (1.4%) and transanal TME (0.7%) were both associated with a lower conversion rate to open surgery compared with laparoscopic TME (8.8%) (*P* < 0.001). Time to flatus and duration of hospital stay were shorter for patients treated with transanal TME (*P* = 0.003 and 0.001 respectively). There were no differences in operating time, intraoperative complications, blood loss, mortality, readmission, R0 resection, or survival.

**Conclusion:**

In this multicentre, retrospective, propensity score-matched, cohort study of patients with locally advanced rectal cancer, newer minimally invasive approaches (robotic TME and transanal TME) demonstrated improved outcomes compared with laparoscopic TME.

## Teaser

The aim of this study was to compare three minimally invasive surgical approaches for total mesorectal excision with primary anastomosis (laparoscopic total mesorectal excision, robotic total mesorectal excision, and transanal total mesorectal excision) using propensity score matching analysis. Newer minimally invasive approaches (robotic total mesorectal excision and transanal total mesorectal excision) demonstrated improved outcomes compared with laparoscopic total mesorectal excision.

## Introduction

Colorectal cancer is the third most diagnosed oncological disease, with rectal cancer accounting for more than 30% of the global diagnoses^1,2^. Despite evolving medical treatments, such as neoadjuvant or adjuvant therapies, surgery remains the mainstay curative option for patients with rectal cancer. Total mesorectal excision (TME) is the standard surgical approach^3^.

Conventionally approached by open surgery, minimally invasive techniques have emerged in the last 20 years as alternatives to open TME, namely laparoscopic, robotic, and transanal surgery for rectal cancer^4^. There are advocates for each of these approaches. However, contrasting evidence, particularly regarding the surgical complication rate and the specimen quality, has generated an unsolved debate about which is the best minimally invasive approach for low/mid locally advanced rectal cancer (LARC).

Laparoscopic TME (L-TME) was proposed as the first minimally invasive approach for rectal cancer treatment^5^. Several trials, such as the MRC-CLASSIC^6^, the COLOR II^7^, and the COREAN^8^ studies, showed no significant difference between open surgery and L-TME in terms of local recurrence and disease-free survival (DFS) rates, supporting the safety and efficacy of laparoscopy for treating LARC in selected patients. However, more recent trials, namely the ACOSOG Z6051^9^ and ALaCaRT^10^ studies, failed to demonstrate the non-inferiority of laparoscopy compared with open surgery concerning pathological outcomes, questioning the oncological safety of L-TME for treating LARC^11^.

As alternatives to laparoscopy, robotic TME (R-TME) and transanal TME (Ta-TME) were introduced as techniques to reduce the technical challenges, while maintaining high-quality oncological outcomes. R-TME was first described in 2006, showing its feasibility in experienced hands^12^. Since then, robotic surgery has become more widespread in the Western world^13,14^, but the efficacy and cost-effectiveness of R-TME as an alternative to L-TME are still debated^15–17^. In 2010, Ta-TME was reported^18^ as a ‘bottom-up’ dissection technique to achieve adequate resection of the distal mesorectum, combining the advantages of transanal dissection with those provided by the minimally invasive approach^19^. Recent meta-analyses, based on retrospective studies and only one RCT^20^, demonstrated similar results in terms of procedural success and postoperative and oncological outcomes of Ta-TME compared with L-TME^21,22^.

The available evidence suggests an evolution in the treatment of low/mid LARC, with a shift toward minimally invasive techniques that are adopted even in the presence of limited or conflicting evidence^23^. To date, there is no RCT comparing the three approaches, namely L-TME, R-TME, and Ta-TME, and only a few retrospective studies have reported comparative analyses in heterogeneous samples of patients with rectal cancer^24–26^.

The aim of this study was to compare the rate of postoperative complications of L-TME *versus* R-TME *versus* Ta-TME using propensity score matching (PSM) on a large multicentre cohort of consecutive patients with low/mid LARC.

## Methods

### Study population

The present study is based on the European MRI and Rectal Cancer Surgery III (EuMaRCS-III) database, which was created by merging prospectively maintained databases of the participating centres. It includes patients with LARC who underwent L-TME, R-TME, or Ta-TME. The European colorectal surgery centres that contributed to the EuMaRCS-III database were: Henri Mondor University Hospital of Créteil, France; Doctor Peset University Hospital of Valencia, Spain; Vall d’Hebron University Hospital of Barcelona, Spain; Fondazione Policlinico Universitario Agostino Gemelli IRCCS, Italy; University Hospital of Padua, Italy; University Hospital of Pisa, Italy; Amsterdam University Medical Centre, The Netherlands; Groupe Hospitalier Diaconesses Croix Saint-Simon, France; and University Hospital of Geneva, Switzerland.

The patient selection criteria were as follows: consecutive patients with histologically proven low/mid LARC (AJCC stages II or III)^27^ treated between January 2013 and January 2022; rectal cancer located within 12 cm from the anal verge; neoadjuvant chemoradiation therapy (NCRT); curative-intent TME procedures with primary anastomosis^28,29^; and pre- and post-NCRT rectal MRI. The patients underwent a long-course of NCRT, delivered in daily fractions of 1.8–2 Gy over a 5- to 6-week interval, with a total radiation dose of 45–50.4 Gy. This was combined with 5-fluorouracil or capecitabine (Xeloda)^30–32^. Records of patients receiving different protocols of NCRT, with synchronous colon cancer, and undergoing abdominoperineal resections or low Hartmann’s procedures with TME were not considered.

All TME operations were performed as standardized procedures^18,33–35^ by experienced senior surgeons (greater than 5 years after the completion of surgical residency) who had completed the learning curve for the surgical technique applied (laparoscopy, robotic, or transanal approach)^36–38^. Procedures performed by junior surgeons were not considered. The participating centres were all high-volume centres; they contributed cases treated using one or more of the three techniques, according to the experience of the centre.

### Study design

The present study was designed as a retrospective, PSM, comparative study. The study protocol was composed before the initiation of the study and sent to the participating centres, some previously involved in the EuMaRCS Study Group^31,39^. The retrospective analyses were conducted on anonymous record data routinely collected in clinical databases. This project complies with the MR004 research category; it was declared to the National Commission for Data Protection and Liberties (CNIL; 2210699) and approved by the Institutional Review Board (00011558). Patients were informed about the research and data were collected and treated in accordance with the ethical standards of the Helsinki Declaration. The STROBE checklist was used for the reporting of the present study^40^ (see the *Supplementary material*).

The present study was designed as a PSM analysis for three groups, as described by Bryer^41^. This method allows the selection bias, inherent in a retrospective study, to be minimized, by considering covariates that may have influenced the selection of the surgical approach used to treat LARC. After PSM, L-TME, R-TME, and Ta-TME were compared with respect to the study outcomes.

### Study outcomes

The main study outcome was the rate of postoperative complications. Secondary outcomes included intraoperative variables (for example conversion rate, operating time, blood loss), postoperative variables (for example duration of hospital stay), the quality of the surgical resection (for example resection margin status, number of harvested lymph nodes), overall survival (OS), and DFS.

Postoperative complications included morbidity and mortality occurring during the hospital stay or within 90 days after surgery. The Dindo–Clavien classification was used to establish the severity of the postoperative complications, with Dindo–Clavien grade greater than or equal to III referring to severe postoperative complications and Dindo–Clavien grade V referring to mortality^42^. Postoperative prolonged ileus was recorded when signs and symptoms of paralytic ileus (absence of bowel movements or flatus, and intolerance of oral intake) persisted for greater than 3 days after surgery^43^. The International Study Group of Rectal Cancer (ISGRC) classification was used to categorize anastomotic leakage^44^.

A premature interruption of the laparoscopic, robotic, or transanal approach before the resection phase was recorded as a converted procedure. The resection was classified as R0 if a macroscopically complete removal of the tumour with a microscopically free resection margin and no peritoneal spread was documented. Disease recurrence was classified as local or distant. Distant metastases were diagnosed either pathologically or on imaging. Local recurrence refers to a tumour deposit located in the pelvic cavity, with pathologically proven adenocarcinoma or objective growth on consecutive imaging^25^.

### Statistical analyses

The statistical analysis was performed using R 4.1.2^45^. Continuous variables are reported as mean(s.d.) and categorical variables are reported as *n* (%). To compare the three surgical procedure groups (L-TME, R-TME, and Ta-TME), chi-squared tests and ANOVA tests were used for categorical and continuous variables respectively. For descriptive purposes, principal component analysis (PCA) was conducted on the variables of interest to graphically display the differences between the three surgical approaches.

The PSM method for the three groups was applied^41^. For each group, propensity scores were calculated using logistic regression models that included the following variables: age, sex, BMI, ASA grade, co-morbidities (greater than 1), tumour size on preoperative CT, tumour category (pT), node category (pN), differentiation grade, and year of surgery. The type of surgical procedure (L-TME *versus* R-TME, L-TME *versus* Ta-TME, and R-TME *versus* Ta-TME) was considered as the dependent variable in the regression model. To minimize the number of retained triplets (that is to reduce the number of duplicate treatment units), the calliper matching method was used. Then, the three matched groups were compared with respect to the study outcomes. Two-by-two comparisons were performed using ANOVA (paired *t* tests) and chi-squared (McNemar) tests. The Benjamini and Yekutieli^46^ correction was applied to all *P* values to adjust for multiple comparisons. For categorical variables that reached statistical significance, ORs and 95% confidence intervals are reported.

The survival analysis was conducted on the whole study sample, as suggested by several studies^47–51^, which demonstrated that studies using Cox regression models can be better at detecting treatment effects than matched studies. OS and DFS (including both local and distant recurrence) were defined as the time from surgery to disease-related death or to disease recurrence respectively. If no event occurred, the patient was censored at the last follow-up date. Survival curves were estimated using Kaplan–Meier methods and a log rank (Mantel–Cox) test was used for group comparisons.

## Results

The study population comprised 468 patients, with a mean(s.d.) age of 64.1(11) years. Overall, 55.1% of the patients had a tumour located in the mid rectum and 44.9% of the patients had a tumour located in the low rectum; 190 (40.6%) patients underwent L-TME, 141 (30.1%) patients underwent R-TME, and 137 (29.3%) patients underwent Ta-TME. *Table 1* displays the baseline characteristics of the total study sample and by surgical approach. Before PSM, the three groups differed significantly in terms of ASA grade, cardiovascular diseases, tumour location, and clinical AJCC stage. The histopathological variables assessed regarding the surgical specimen were statistically different for ypN stage, lymphovascular invasion, perineural invasion, tumour deposit, tumour grade, and use of adjuvant treatment.

**Table 1:** Demographic, clinical, and histological/oncological characteristics of patients with locally advanced rectal cancer who underwent laparoscopic total mesorectal excision, robotic total mesorectal excision, or transanal total mesorectal excision

	Whole sample (*n* = 468)	L-TME (*n* = 190)	R-TME (*n* = 141)	Ta-TME (*n* = 137)	*P*
**Demographic and clinical variables**					
Sex					0.718
Male	296	120	86	90	
Female	172	70	55	47	
Age (years), mean(s.d.)	64.1(11)	64.9(10.8)	64.1(11.2)	63.0(10.8)	0.317
Age >75 years	69 (14.7)	33 (17.4)	18 (12.8)	18 (13.1)	0.414
BMI (kg/m^2^), mean(s.d.)	25.6(4.5)	25.9(4.4)	25.5(4.2)	25.3(4.6)	0.457
Obesity (BMI ≥30 kg/m^2^)	73 (15.6)	31 (16.3)	22 (15.6)	20 (14.6)	0.914
ASA grade					<0.0001*
I–II	376 (80.3)	143 (75.3)	107 (75.9)	126 (92)	
III–IV	92 (19.7)	47 (24.7)	34 (24.1)	11 (8)	
Preoperative serum CEA (U/mL), mean(s.d.)	9.7(24.7)	10.5(25.3)	9.6(23.6)	8.8(25.0)	0.821
Albumin serum level (g/L), mean(s.d.)	40.1(5.9)	40.1(6.3)	39.4(6.1)	40.8(5.1)	0.150
Preoperative leucocytes (10^9^/L), mean(s.d.)	5.8(1.9)	5.8(2.0)	5.9(1.7)	5.8(2.0)	0.853
Co-morbidities (>1)	133 (28.4)	56 (29.5)	37 (26.2)	40 (29.2)	0.768
Diabetes	53 (11.3)	24 (12.6)	15 (10.6)	14 (10.2)	0.757
Cardiovascular diseases	181 (38.7)	53 (27.9)	69 (48.9)	59 (43.1)	<0.0001*
Pulmonary diseases	44 (9.4)	20 (10.5)	14 (9.9)	10 (7.3)	0.594
Kidney failure	16 (3.4)	9 (4.7)	5 (3.5)	2 (1.5)	0.272
Neurocognitive disorders	7 (1.5)	3 (1.6)	2 (1.4)	2 (1.5)	0.992
Smoking	142 (30.3)	51 (26.8)	41 (29.1)	50 (36.5)	0.160
Previous abdominal surgery	168 (35.9)	71 (37.4)	49 (34.7)	48 (35)	0.859
Tumour location					<0.0001*
Mid rectum (5 to <10 cm)	258 (55.1)	134 (70.5)	48 (34.0)	76 (55.5)	
Low rectum (<5 cm)	210 (44.9)	56 (29.5)	93 (66.0)	61 (44.5)	
Clinical stage of disease (AJCC)					<0.0001*
II	99 (21.2)	20 (10.5)	61 (43.3)	18 (13.1)	
III	369 (78.8)	170 (89.5)	80 (56.7)	119 (86.9)	
**Histological/oncological variables**					
ypT stage					0.248
0	108 (23.1)	49 (25.8)	32 (22.7)	26 (19)	
1	34 (7.3)	14 (7.4)	9 (6.4)	11 (8)	
2	122 (26.1)	49 (25.8)	36 (25.5)	37 (27)	
3	189 (40.4)	72 (37.9)	56 (39.7)	61 (44.5)	
4a	9 (1.9)	3 (1.6)	5 (3.5)	1 (0.7)	
4b	6 (1.3)	3 (1.6)	3 (2.1)	0	
ypN stage					0.012*
0	358 (76.5)	159 (83.7)	99 (70.2)	100 (73)	
1	85 (18.2)	21 (11)	32 (22.7)	32 (23.3)	
2	25 (5.3)	10 (5.3)	10 (7.1)	5 (3.6)	
Tumour sterilization ypT0 N0	96 (20.5)	47 (24.7)	28 (19.9)	21 (15.3)	0.112
Lymph vascular invasion	63 (13.5)	36 (18.9)	13 (9.2)	14 (10.2)	0.016*
Perineural invasion	40 (8.5)	27 (14.2)	5 (3.5)	8 (5.8)	0.001*
Tumour size (largest dimension, cm), mean(s.d.)	2.1(1.7)	2.0(1.6)	2.3(1.7)	2.1(1.7)	0.172
Tumour deposit	74 (15.8)	55 (28.9)	7 (5.0)	12 (8.8)	<0.0001*
Tumour grade					<0.0001*
Well differentiated	212 (45.3)	78 (41)	70 (49.6)	64 (46.7)	
Moderately differentiated	202 (43.2)	76 (40)	59 (41.9)	67 (48.9)	
Poorly differentiated	53 (11.3)	36 (19)	12 (8.5)	6 (4.4)	
Adjuvant treatment	204 (43.6)	102 (53.7)	52 (36.9)	50 (36.5)	0.001*

Values are *n* (%) unless otherwise indicated. L-TME, laparoscopic total mesorectal excision; R-TME, robotic total mesorectal excision; Ta-TME, transanal total mesorectal excision; CEA, carcinoembryonic antigen. *Statistically significant.

**Fig. 1:**
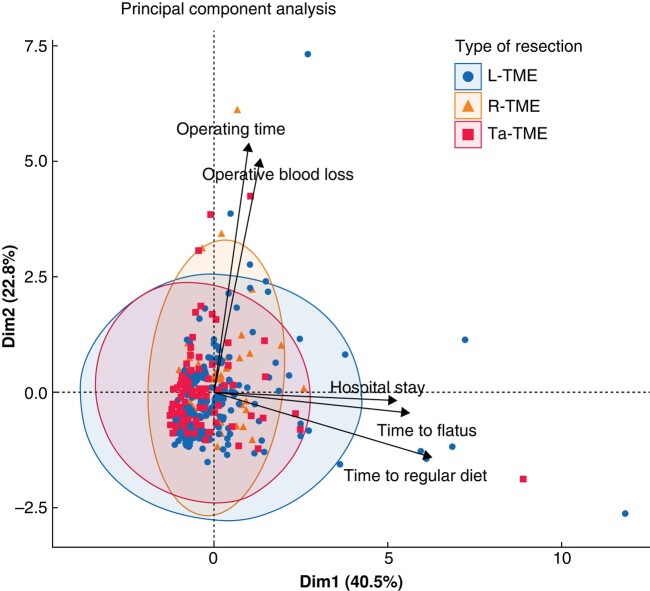
Principal component analysis for the three types of surgical approaches (laparoscopic total mesorectal excision, robotic total mesorectal excision, and transanal total mesorectal excision) L-TME, laparoscopic total mesorectal excision; R-TME, robotic total mesorectal excision; Ta-TME, transanal total mesorectal excision.

Explorative analysis by PCA (*Fig. 1*) showed three clusters related to L-TME, R-TME, and Ta-TME that explained 63.3% of the total variability on the first two axes. The three groups varied in terms of operating time and operative blood loss (*x*-axis) and postoperative recovery and hospital stay (*y*-axis) before PSM.

After PSM, each group comprised 148 patients. The L-TME, R-TME, and Ta-TME groups were well balanced in terms of the variables included in the PSM model and for most of the demographic characteristics (*Fig. S1* and *Table S1*). The between-group comparisons in terms of operative and postoperative outcomes are presented in *Table 2*. No significant difference was found for the mean operating time, which ranged between 289.5 min for L-TME and 309.1 min for R-TME. Similarly, no differences were observed in terms of the rate of intraoperative complications, which occurred in 4.7% of patients in the R-TME and Ta-TME groups and in 10.8% of patients in the L-TME group (*P* = 0.055). Operative blood loss and the need for blood transfusion were not significantly different between the groups, although they were inferior for Ta-TME. A significantly lower conversion rate to laparotomy was found for R-TME (1.4%) and Ta-TME (0.7%) compared with L-TME (8.8%), with no statistically significant difference between R-TME and Ta-TME. No conversion from R-TME to L-TME occurred. Additionally, the rate of postoperative complications was significantly lower for R-TME and Ta-TME than for L-TME, with ORs of 1.80 and 2.87 for L-TME compared with R-TME and Ta-TME respectively. According to the ISGRC classification for anastomotic leakage, 8.8% of patients in the L-TME group had a grade A anastomotic leakage *versus* 2% of patients in the R-TME group and 8.1% of patients in the Ta-TME group (*P* = 0.031). The post-hoc analysis showed that the R-TME approach was protective in terms of grade A anastomotic leakage compared with both L-TME (OR 0.21, 95% c.i. 0.06–0.70) and Ta-TME (OR 0.23, 95% c.i. 0.06–0.84). No statistically significant difference was noted between L-TME and Ta-TME for grade A anastomotic leakage. Conversely, there was a higher rate of grade C anastomotic leakage in the Ta-TME group (4.1% *versus* 0.7% in the R-TME group and 0 in the L-TME group).

**Table 2:** Operative and postoperative outcomes of patients with locally advanced rectal cancer after propensity score matching

Variables	L-TME (*n* = 148)	R-TME (*n* = 148)	Ta-TME (*n* = 148)	Overall comparison, *P*	Two-by-two comparisons, *P* and/or OR (95% c.i.)		
					L-TME *versus* R-TME	L-TME *versus* Ta-TME	R-TME *versus* Ta-TME
Operating time (min), mean(s.d.)	289.5(83)	309.1(86.9)	300(74.1)	0.118	–	–	–
Conversion to laparotomy	13 (8.8)	2 (1.4)	1 (0.7)	<0.001*	0.01* and 7.02 (1.56–31.72)*	0.003* and 14.15 (1.82–109.67)*	1
Operative blood loss (mL), mean(s.d.)	98.9(163)	104.5(130.3)	82.2(114.4)	0.348	–	–	–
Intraoperative complication	16 (10.8)	7 (4.7)	7 (4.7)	0.055	–	–	–
Patients with postoperative complication	64 (43.2)	44 (29.7)	31 (20.9)	<0.001*	1.80 (1.11–2.91)*	2.87 (1.72–4.80)*	1.60 (0.94–2.71)
Postoperative blood transfusion	8 (5.4)	5 (3.4)	1 (0.7)	0.065	–	–	–
**ISGRC anastomotic leakage**							
A	13 (8.8)	3 (2)	12 (8.1)	0.031*	4.65 (1.29–16.69)*	1.09 (0.48–2.48)	0.23 (0.06–0.84)*
B	5 (3.4)	3 (2)	0	0.089	–	–	–
C	0	1 (0.7)	6 (4.1)	0.011*	0	0	0.17 (0.01–1.41)
Prolonged ileus	20 (13.5)	6 (4.1)	9 (6.1)	0.006*	3.70 (1.40–9.50)*	2.40 (1.10–5.50)*	0.7 (0.2–1.9)
Postoperative complication classified as Dindo–Clavien grade ≥III†	20 (32.8)	15 (32.6)	10 (32.2)	0.857	–	–	–
Reoperation	18 (12.2)	11 (7.4)	10 (6.8)	0.201	–	–	–
Time to flatus, mean(s.d.)	2.1(1.4)	1.7(0.9)	1.7(1.5)	0.052	–	–	–
Return to regular diet, mean(s.d.)	3.3(2.3)	3.3(1.6)	2.6(2.2)	0.003*	0.876	0.008*	0.0013*
Duration of hospital stay (days)‡, mean(s.d.)	11.7(6.5)	12.3(10.2)	7.1(6)	0.001*	0.511	<0.001*	<0.001*
Mortality at 90 days	0	1 (0.7)	0	–	–	–	–
Readmission within 60 days	10 (6.8)	4 (2.7)	13 (8.8)	0.083	–	–	–
R1 status	13 (8.8)	13 (8.8)	5 (3.4)	0.108	–	–	–
Positive circumferential resection margin	11 (7.4)	13 (8.8)	5 (3.4)	0.146	–	–	–
Positive distal resection margin	3 (2)	3 (2)	0	0.218	–	–	–
Harvested lymph nodes, mean(s.d.)	16.4(10)	15.1(6.4)	12.9(5.7)	<0.001*	0.169	<0.001*	0.002*
Harvested lymph nodes ≥12	98 (66.2)	107 (72.3)	86 (58.1)	0.036*	0.75 (0.45–1.23)	1.41 (0.88–2.26)	1.88 (1.6–3.05)
Stoma closure	81 (60)	120 (91.6)	117 (81.8)	<0.001*	<0.001* and 0.14 (0.07–0.27)	<0.001* and 0.33 (0.19–0.58)	0.018* and 2.42 (1.15–5.13)

Values are *n* (%) unless otherwise indicated. *Statistically significant. †Calculated for patients with postoperative complications. ‡Excluding deceased patients. L-TME, laparoscopic total mesorectal excision; R-TME, robotic total mesorectal excision; Ta-TME, transanal total mesorectal excision; ISGRC, International Study Group of Rectal Cancer.

The rate of prolonged ileus was higher for the L-TME group (13.5%), with an increased OR compared with both R-TME (OR 3.70, 95% c.i. 1.40–9.50) and Ta-TME (OR 2.40, 95% c.i. 1.10–5.50), with no difference between R-TME and Ta-TME. However, there was no between-group difference in terms of the rate of severe postoperative complications (Dindo–Clavien grade greater than or equal to III). The mean time to return to a regular diet was 3.3 days for L-TME, 3.3 days for R-TME, and 2.6 days for Ta-TME, with a significant difference in favour of Ta-TME compared with the other two groups. The same was noted for the mean duration of hospital stay, which was shorter for patients in the Ta-TME group. There was no 90-day mortality in the L-TME group or the Ta-TME group and the 90-day mortality rate for the R-TME group was 0.7%. No group-related difference was observed for R1 status, positive circumferential resection margin (CRM), or positive distal resection margin (DRM). The mean number of harvested lymph nodes was higher in the L-TME and R-TME groups than in the Ta-TME group.

Data regarding the quality of the mesorectal excision were not available for the entire sample. Complete excision was achieved for 78.3% of L-TME cases, 97.3% of R-TME cases, and 82.4% of Ta-TME cases (overall *P* < 0.001). Two-by-two comparisons were not performed due to missing data, particularly in the Ta-TME group; no statistical imputation was conducted due to the characteristics of the outcome. Stoma closure was more frequently performed after R-TME (91.6% of the patients) and after Ta-TME (81.8% of the patients) than after L-TME (60% of the patients).

**Fig. 2:**
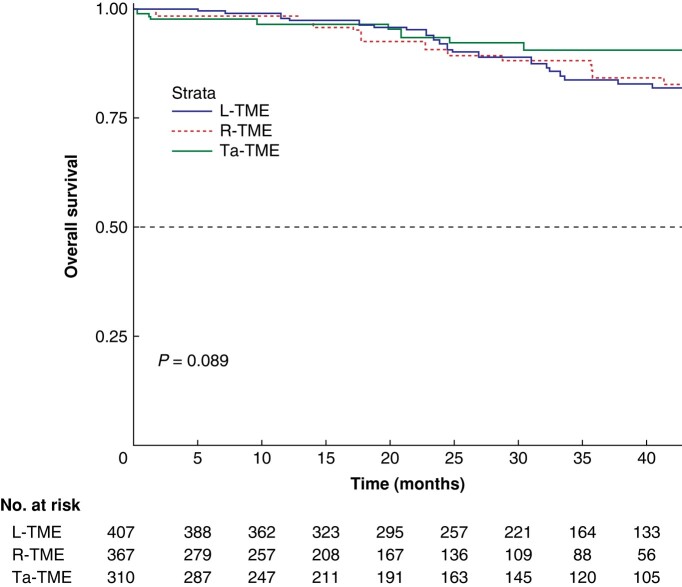
Kaplan–Meier curve for overall survival The 1-, 2-, and 5-year overall survival rates were 97.8%, 92.1%, and 69.4% respectively for the laparoscopic total mesorectal excision group, 98.4%, 90.7%, and 82.8% respectively for the robotic total mesorectal excision group, and 96.4%, 93.4%, and 90.5% respectively for the transanal total mesorectal excision group (*P* = 0.089). L-TME, laparoscopic total mesorectal excision; R-TME, robotic total mesorectal excision; Ta-TME, transanal total mesorectal excision.

**Fig. 3:**
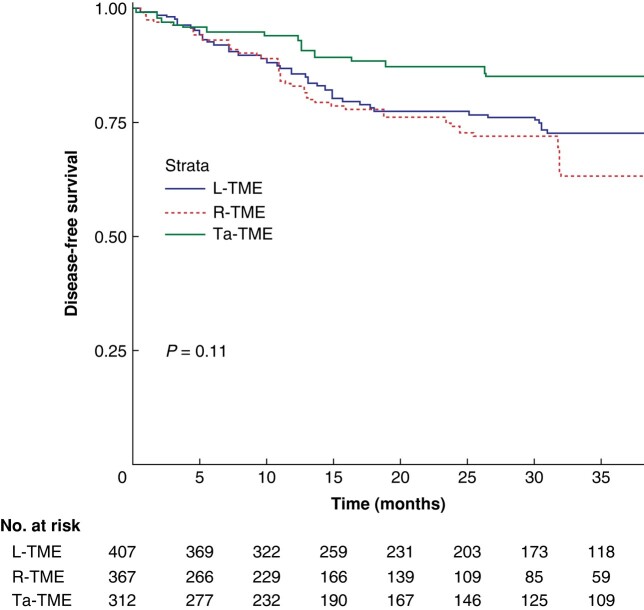
Kaplan–Meier curve for disease-free survival The 1-, 2-, and 5-year DFS rates were 85.6%, 77.5%, and 72.8% respectively for the laparoscopic total mesorectal excision group, 83%, 74.1%, and 63.3% respectively for the robotic total mesorectal excision group, and 94%, 82.7%, and 69.6% respectively for the transanal total mesorectal excision group (*P* = 0.11). L-TME, laparoscopic total mesorectal excision; R-TME, robotic total mesorectal excision; Ta-TME, transanal total mesorectal excision.

The survival analysis was performed on the whole study sample and is shown in *Figs. 2* and *3* for OS and DFS respectively. No between-group differences were found. The multivariate Cox regression models of covariates predicting OS and DFS showed that age (HR 1.07, 95% c.i. 1.03–1.10; *P* < 0.001), tumour size (HR 1.27, 95% c.i. 1.13–1.44; *P* < 0.001), and R1 status (HR 7.00, 95% c.i. 3.20–15.26; *P* < 0.001) were significantly associated with OS, whereas ypT stage greater than 2 (HR 2.88, 95% c.i. 1.45–3.58; *P* < 0.001) was significantly associated with DFS.

## Discussion

Due to the paucity of literature comparing L-TME, R-TME, and Ta-TME, this PSM analysis adds further data on the role of minimally invasive approaches for the resection of LARC. In a relatively large and homogeneous cohort of patients with low/mid LARC, L-TME was associated with a higher conversion rate to open surgery, a higher rate of postoperative complications, and a reduced rate of stoma closure compared with both R-TME and Ta-TME. Compared with R-TME and L-TME, Ta-TME was associated with a significantly shorter time to return to a regular diet and a shorter hospital stay. However, a significantly lower number of retrieved lymph nodes was found in the Ta-TME group, with only 58.1% of procedures associated with greater than or equal to 12 harvested lymph nodes. R-TME showed the lowest rate of grade A anastomotic leakage compared with both L-TME and Ta-TME. No difference was noted for OS and DFS between the three surgical approaches.

L-TME remains the most popular minimally invasive approach for rectal cancer. In a nationwide study conducted in Denmark, 48% of all TME operations were approached laparoscopically, compared with 13% transanally and 29.8% robotically^26^. The predominant role of laparoscopy is likely to be the consequence of its earlier introduction and widespread adoption. However, laparoscopy is considered a highly challenging technique for treating LARC^31,39^. R-TME and Ta-TME have been seen as valuable alternatives to overcome the technical limitations of L-TME. However, they are still applied on an empirical basis, mainly related to surgeons’ preferences and experience. It is not surprising that, when comparing the entire study population that underwent L-TME, R-TME, or Ta-TME (before PSM), several significant differences were noted in the demographic and clinical characteristics.

The use of R-TME has been associated with potential technical advantages^52^. The ROLARR multicentre international trial^15^, comparing L-TME and R-TME performed by surgeons with various degrees of experience in robotic surgery, aimed to evaluate the safety and efficacy of R-TME, particularly with regard to reducing the risk of conversion to open surgery. The results showed no significant differences in terms of conversion rate and pathological outcomes, and Jayne *et al*.^15^ concluded that R-TME does not confer an advantage in rectal cancer resection compared with L-TME. A more recent multicentre RCT, the REAL trial, showed that R-TME performed by experienced surgeons (who had completed the learning curve) results in better short-term outcomes than L-TME, with less surgical trauma, better postoperative recovery, and better oncological quality of resection (complete resection)^16^. However, it must be noted that the REAL trial was designed with a primary outcome of 3-year locoregional recurrence, which has not yet been reached. The published results represent secondary endpoints; moreover, caution should also be paid regarding the interpretation of the oncological quality of the resection (CRM less than or equal to 1 mm), which does not linearly and directly translate into worse long-term oncological outcomes or poorer survival.

The role of Ta-TME is highly debated in the literature. Nationwide, a letter of warning was published by Larsen *et al*.^53^ after the observation of a 9.5% rate of early local recurrence with an unexpected pattern after the implementation of Ta-TME in Norway. This moratorium raised attention toward the safety of Ta-TME, but the Norwegian audit aiming to provide the detailed reasons behind this high rate of early local recurrence, as well as about the quality of the mesorectum and the resection margins, has not yet been published. Ta-TME is currently under investigation by three ongoing trials: the COLOR III^54^, the GRECCAR 11^55^, and the TaLaR^56^ studies, which are exploring the pathological and oncological results of Ta-TME compared with L-TME. The preliminary results are encouraging^57^.

With contrasting evidence and no available RCT comparing the three minimally invasive approaches for TME, retrospective PSM studies can provide evidence to support clinical practice and further research. Based on the present results, R-TME and Ta-TME are associated with better short-term outcomes than L-TME. A significantly lower conversion rate to open surgery was observed for R-TME (1.4%) and Ta-TME (0.7%) than for L-TME (8.8%). The observed low conversion rates are similar to those of the retrospective study by Hol *et al*.^24^, which compared three PS-matched groups of 108 patients operated on by experienced surgeons only. However, Hol *et al*.^24^ found no significant difference in the conversion rate between L-TME, R-TME, and Ta-TME. In the ROLARR trial, conversion to laparotomy was the primary outcome and the reported rates were 12.2% for laparoscopy and 8.1% for robot-assisted surgery, but no statistically significant difference was found. Conversely, in the REAL trial, a significantly lower conversion rate was observed for R-TME (1.7%) compared with L-TME (3.9%)^16^. More recently, a network meta-analysis estimated a risk ratio for conversion of 0.23 (95% credible interval 0.034 to 0.7) for R-TME compared with L-TME^58^; the risk ratio was also in favour of Ta-TME compared with L-TME, although it did not reach statistical significance^58^. Ta-TME was also associated with the lowest conversion rate in the nationwide study of Ose *et al*.^26^, who reported rates of 10.9% for L-TME, 5.6% for R-TME, and 1.3% for Ta-TME (*P* < 0.0001). Despite the difference in the study design (prospective *versus* retrospective respectively), the literature and the present results support the feasibility and applicability of R-TME and Ta-TME in resecting LARC as they are associated with a low risk of conversion to open surgery. This is clinically meaningful, as patients converted from a minimally invasive approach to open surgery were found to be at higher risk of perioperative mortality and morbidity and to have worse oncological outcomes^6,59^, endorsing any effort made to anticipate surgical difficulties and prevent conversion.

In the present study, the rate of postoperative complications (within 90 days) was significantly higher for the L-TME group than for both the R-TME group (OR 1.80) and the Ta-TME group (OR 2.87). In particular, the rates of prolonged postoperative ileus and grade A anastomotic leakage were higher in the L-TME group than in the R-TME and Ta-TME groups. However, Ta-TME was associated with more grade C anastomotic leakage than the two other approaches. The observed morbidity rates are in line with previous studies^4,16,24,26,58^, but not all had a significant difference between the groups. This may be related to the difference in sample size and study power, but also to the definition and classification applied for postoperative complications. According to the most commonly used Dindo–Clavien classification, no between-group difference was observed for severe postoperative morbidity or mortality. Conversely, the significantly lower rate of grade A anastomotic leakage in R-TME (2% *versus* 8.1% for Ta-TME and 8.1% for L-TME) is consistent with a recent network meta-analysis that demonstrated, based on 23 studies including 1193 L-TME, 1302 R-TME, and 508 Ta-TME cases, that L-TME was associated with a higher risk of anastomotic leakage (risk ratio 1.4, 95% credible interval 1.1 to 1.9) than R-TME, also ranking first in the empirical probabilities between the surgical approaches, based on indirect evidence^60^. Interestingly, in the present study, the highest rate of stoma closure occurred in the R-TME group, in which the lowest rate of anastomotic leakage occurred.

Postoperative recovery, namely the time to return to a regular diet and the duration of hospital stay, was shorter in the Ta-TME group than in the L-TME and R-TME groups. This is in line with a recent meta-analysis that, based on indirect evidence, ranked Ta-TME as the first (best), R-TME as the second, L-TME as the third, and open TME as the last (worst) surgical approach concerning the total duration of hospital stay^4^. However, in the present study, the number of harvested lymph nodes was lowest in the Ta-TME group, being significantly inferior to the R-TME and L-TME groups. When evaluating the number of patients for whom at least 12 lymph nodes were harvested, a significant difference was observed only between R-TME and Ta-TME, in favour of R-TME, with greater than or equal to 12 lymph nodes retrieved in 72% of patients *versus* 58% of patients respectively. Despite this finding, the other pathological outcomes, namely the R0 resection rate, the rates of positive CRM and DRM, and the rate of complete mesorectal excision, were similar between the groups, as were the OS and DFS rates. Previous studies assessing pathological outcomes and survival between L-TME and R-TME reported similar results^15–17,60^. Only a few observational studies have compared R-TME and Ta-TME, showing no difference in terms of pathological results^61,62^. The observational ROTA trial is currently enrolling patients with the same comparative purpose^63^. The network meta-analysis by Rausa *et al*.^60^, focusing on pathological outcomes, concluded that the three approaches have comparable results regarding mesorectum excision quality and local and distant recurrence. Burghgraef *et al*.^25^ analysed the 3-year oncological results of L-TME, R-TME, and Ta-TME in a population-based cohort of 617 patients from experienced surgical centres and showed similar results for OS and DFS, which were not different between the groups. Burghgraef *et al*.^25^ conducted a multivariate Cox regression analysis that did not show any significant difference between the three surgical approaches, while taking confounders into account. Additionally, in the present study, the Cox regression models showed that the surgical approach is not predictive of OS and DFS.

The present study has some limitations. This is a retrospective study, based on prospectively maintained databases. Although PSM allowed minimization of potential selection bias, the presence of residual confounders cannot be ruled out. The surgical procedures and follow-up were all performed by experienced surgeons in university hospitals; thus, caution should be paid when generalizing the results to other types of institutions and populations. The impact of the learning curve for minimally invasive techniques, such as robotic and transanal approaches, is not negligible^15,16,24,64^ and should always be considered when comparing the present data with the available literature.

## Supplementary Material

zrae044_Supplementary_Data

## Data Availability

The data presented in this study are available on request from the corresponding author.

## Collaborators

### European MRI and Rectal Cancer Surgery (EuMaRCS) Study Group

Giorgio Bianchi (Beaujon University Hospital (AP-HP), Clichy, France; University Paris Cité, Paris, France); Eva Martí-Martínez (Hospital Universitario Doctor Peset, Valencia, Spain); Teresa Torres-Sánchez (Hospital Universitario Doctor Peset, Valencia, Spain); Juan Carlos Sebastián-Tomás (Hospital Universitario Doctor Peset, Valencia, Spain); Isacco Maretto (University of Padua, Padua, Italy); Gaya Spolverato (University of Padua, Padua, Italy); Simone Guadagni (University of Pisa, Pisa, Italy); Alejandro Solis (University Hospital Vall d’Hebron, Universitat Autonoma de Barcelona, Barcelona, Spain).
