# Supplementary material for: Robotic, transanal, and laparoscopic total mesorectal excision for locally advanced mid/low rectal cancer: European multicentre, propensity score-matched study
Source: BJS Open. 2024 May 28;8(3):zrae044. doi: 10.1093/bjsopen/zrae044 (PMC11132137; doi:10.1093/bjsopen/zrae044)
Supplement: zrae044_Supplementary_Data [file zrae044_supplementary_data.docx]

**Robotic, transanal, and laparoscopic total mesorectal excision for locally advanced mid-low rectal cancer: a European multicentre, propensity score-matched study**

Authors: Nicola de’Angelis^1^, Francesco Marchegiani^2^, Aleix Martínez-Pérez^3,4^, Alberto Biondi^5^, Salvatore Pucciarelli^6^, Carlo Alberto Schena^1,*^, Gianluca Pellino^7^, Miquel Kraft^7^, Annabel S. van Lieshout^8^, Luca Morelli^9^, Alain Valverde^10^, Renato Micelli Lupinacci^10^, Segundo A. Gómez-Abril^3^, Roberto Persiani^5^, Jurriaan B Tuynman^8^, Eloy Espin-Basany^7^, Frederic Ris^11^, on behalf of the EuMaRCS Study Group

Collaborators (EuMaRCS Study Group): Giorgio Bianchi^2^, Eva Martí-Martínez^3^, Teresa Torres-Sánchez^3^, Juan Carlos Sebastián-Tomás^3^, Isacco Maretto^6^, Gaya Spolverato^6^, Simone Guadagni^9^ and Alejandro Solis^7^.

^1^ Unit of Robotic and Minimally Invasive Digestive Surgery, Department of Surgery, Ferrara University Hospital, Ferrara (Cona), Italy.

^2^ Unit of Colorectal and Digestive Surgery, DIGEST Department, Beaujon University Hospital (AP-HP), Clichy, France and University Paris Cité, Paris, France.

^3^ Unit of Colorectal Surgery, Department of General and Digestive Surgery, Hospital Universitario Doctor Peset, Valencia, Spain.

^4^ Biosanitary Research Institute. Valencian International University (VIU), Valencia, Spain.

^5^ General Surgery Unit, Fondazione Policlinico Universitario Agostino Gemelli IRCCS, Rome, Italy.

^6^ General Surgery 3, Department of Surgery, Oncology and Gastroenterology, University of Padova, Padova, Italy.

^7^ Unit of Colorectal Surgery, Department of General and Digestive Surgery, University Hospital Vall d’Hebron, Barcelona, Universitat Autonoma de Barcelona, Spain.

^8^ Department of Surgery, Amsterdam University Medical Center, Location Vrije Universiteit Amsterdam, Amsterdam, The Netherlands.

^9^ General Surgery Unit, Department of Translational Research and New Technologies in Medicine and Surgery, University of Pisa, Pisa, Italy.

^10^ Department of Digestive Surgery, Groupe Hospitalier Diaconesses, Croix Saint-Simon, 75020, Paris, France.

^11^ Service of Abdominal Surgery, Geneva University Hospitals and Medical School, Geneva, Switzerland.

**Corresponding authors**

Dr Carlo Alberto Schena, carloalbertoschena@gmail.com, **ORCID ID: 0000-0003-1136-1103**; **Twitter: @CA_Schena**

Prof Nicola de’Angelis, nic.deangelis@yahoo.it, **ORCID ID: 0000-0002-1211-4916**; **Twitter: NdeA81**

**Supplementary Materials - Index**

| **Supplementary Methods** |  |
| --- | --- |
| NA |  |
| **Supplementary Results** |  |
| NA |  |
| **Supplementary Appendixes** |  |
| STROBE Checklist | *page 3* |
| **Supplementary Figures and Tables** |  |
| Supplementary Figure 1 | *page 6* |
| Supplementary Table 1 | *Page 7* |
|  |  |

**Supplementary Appendixes**

**Supplemental Table 1.** STROBE checklist for the study: “Robotic, transanal, and laparoscopic total mesorectal excision for locally advanced mid-low rectal cancer: European multicentre, propensity score-matched study”.

|  | Item No. | Recommendation | Page  No. |
| --- | --- | --- | --- |
| **Title and abstract** | 1 | (*a*) Indicate the study’s design with a commonly used term in the title or the abstract | 1, 4 |
|  |  | (*b*) Provide in the abstract an informative and balanced summary of what was done and what was found | 4 |
| Introduction | | | |
| Background/rationale | 2 | Explain the scientific background and rationale for the investigation being reported | 6 |
| Objectives | 3 | State specific objectives, including any prespecified hypotheses | 7 |
| Methods | | | |
| Study design | 4 | Present key elements of study design early in the paper | 8 |
| Setting | 5 | Describe the setting, locations, and relevant dates, including periods of recruitment, exposure, follow-up, and data collection | 8 |
| Participants | 6 | (*a*) *Cohort study*—Give the eligibility criteria, and the sources and methods of selection of participants. Describe methods of follow-up  *Case-control study*—Give the eligibility criteria, and the sources and methods of case ascertainment and control selection. Give the rationale for the choice of cases and controls  *Cross-sectional study*—Give the eligibility criteria, and the sources and methods of selection of participants | 8, 9 |
|  |  | (*b*) *Cohort study*—For matched studies, give matching criteria and number of exposed and unexposed  *Case-control study*—For matched studies, give matching criteria and the number of controls per case | 11 |
| Variables | 7 | Clearly define all outcomes, exposures, predictors, potential confounders, and effect modifiers. Give diagnostic criteria, if applicable | 9, 10 |
| Data sources/ measurement | 8* | For each variable of interest, give sources of data and details of methods of assessment (measurement). Describe comparability of assessment methods if there is more than one group | 10 |
| Bias | 9 | Describe any efforts to address potential sources of bias | 11 |
| Study size | 10 | Explain how the study size was arrived at | 11 |

Continued on next page

| Quantitative variables | 11 | Explain how quantitative variables were handled in the analyses. If applicable, describe which groupings were chosen and why | 11 |
| --- | --- | --- | --- |
| Statistical methods | 12 | (*a*) Describe all statistical methods, including those used to control for confounding | 10, 11 |
|  |  | (*b*) Describe any methods used to examine subgroups and interactions | 11 |
|  |  | (*c*) Explain how missing data were addressed | 11 |
|  |  | (*d*) *Cohort study*—If applicable, explain how loss to follow-up was addressed  *Case-control study*—If applicable, explain how matching of cases and controls was addressed  *Cross-sectional study*—If applicable, describe analytical methods taking account of sampling strategy | 11 |
|  |  | (*e*) Describe any sensitivity analyses | NA |
| **Results** |  |  |  |
| Participants | 13* | (a) Report numbers of individuals at each stage of study—eg numbers potentially eligible, examined for eligibility, confirmed eligible, included in the study, completing follow-up, and analysed | 12 |
|  |  | (b) Give reasons for non-participation at each stage | NA |
|  |  | (c) Consider use of a flow diagram | NR |
| Descriptive data | 14* | (a) Give characteristics of study participants (eg demographic, clinical, social) and information on exposures and potential confounders | 12, TABLE 1, FIG 1 |
|  |  | (b) Indicate number of participants with missing data for each variable of interest | - |
|  |  | (c) *Cohort study*—Summarise follow-up time (eg, average and total amount) | 10-12 |
| Outcome data | 15* | *Cohort study*—Report numbers of outcome events or summary measures over time | TABLE 2 |
|  |  | *Case-control study—*Report numbers in each exposure category, or summary measures of exposure |  |
|  |  | *Cross-sectional study—*Report numbers of outcome events or summary measures |  |
| Main results | 16 | (*a*) Give unadjusted estimates and, if applicable, confounder-adjusted estimates and their precision (eg, 95% confidence interval). Make clear which confounders were adjusted for and why they were included | 13, TABLE 2 |
|  |  | (*b*) Report category boundaries when continuous variables were categorized | TABLES |
|  |  | (*c*) If relevant, consider translating estimates of relative risk into absolute risk for a meaningful time period | NA |

Continued on next page

| Other analyses | 17 | Report other analyses done—eg analyses of subgroups and interactions, and sensitivity analyses |  | 14 |
| --- | --- | --- | --- | --- |
| Discussion | | | | |
| Key results | 18 | Summarise key results with reference to study objectives |  | 15 |
| Limitations | 19 | Discuss limitations of the study, taking into account sources of potential bias or imprecision. Discuss both direction and magnitude of any potential bias |  | 19, 20 |
| Interpretation | 20 | Give a cautious overall interpretation of results considering objectives, limitations, multiplicity of analyses, results from similar studies, and other relevant evidence |  | 16, 17, 18 |
| Generalisability | 21 | Discuss the generalisability (external validity) of the study results |  | 20 |
| Other information | |  | | |
| Funding | 22 | Give the source of funding and the role of the funders for the present study and, if applicable, for the original study on which the present article is based |  | 2 |

*Give information separately for cases and controls in case-control studies and, if applicable, for exposed and unexposed groups in cohort and cross-sectional studies.

**Note:** An Explanation and Elaboration article discusses each checklist item and gives methodological background and published examples of transparent reporting. The STROBE checklist is best used in conjunction with this article (freely available on the Web sites of PLoS Medicine at http://www.plosmedicine.org/, Annals of Internal Medicine at http://www.annals.org/, and Epidemiology at http://www.epidem.com/). Information on the STROBE Initiative is available at www.strobe-statement.org.

**Supplementary Figures and Tables**

**Supplementary Figure** **1**. Multiple covariate balance plot of absolute standardized effect sizes before and after propensity score matching.

**
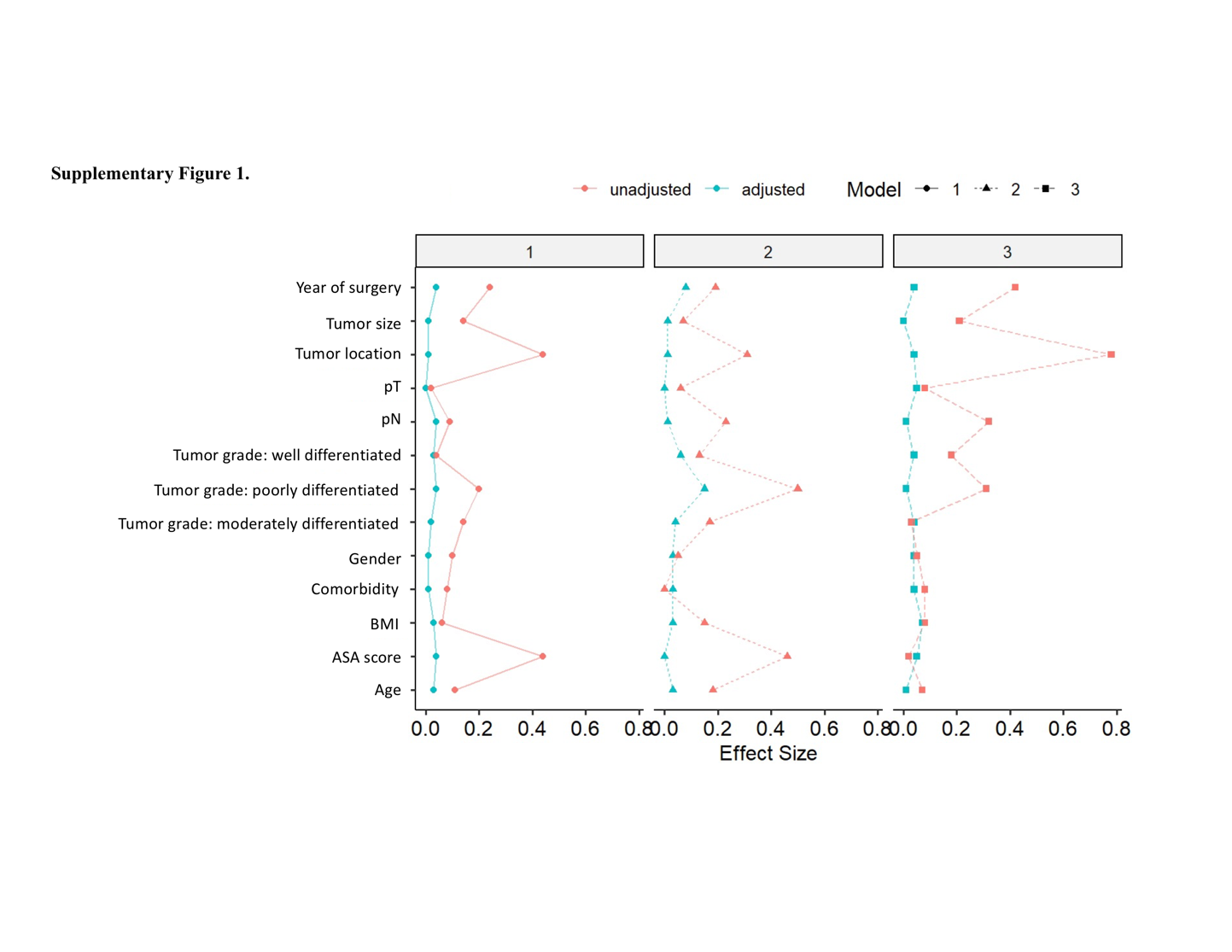
**

**Supplemental Table 1. Demographic data of patients with LARC after propensity score matching.**

|  | L-TME  (n=148) | R-TME  (n=148) | TaTME  (n=148) | P value |
| --- | --- | --- | --- | --- |
| *Demographic and clinical variables* |  |  |  |  |
| Gender (M/F) [n] | 100 / 48 | 95 / 53 | 103 / 45 | 0.607 |
| Age (yr) [mean (SD)] | 65.7 (10.8) | 63.7 (10.8) | 63.7 (10.1) | 0.161 |
| BMI (kg/m^2^) [mean (SD)] | 25.6 (4.4) | 24.8 (4.9) | 25.0 (4.0) | 0.264 |
| ASA score [n%]   - I-II - III-IV | 135 (91.2)  13 (8.8) | 131 (88.5)  17 (11.5) | 138 (93.2)  10 (6.8) | 0.362 |
| Preoperative serum CEA (U/mL) [mean (SD)] | 10 (23) | 7.3 (13.3) | 8.9 (24.1) | 0.529 |
| Albumin serum level (g/L) [mean (SD)] | 40.6 (5.5) | 39.2 (5.9) | 41.3 (5.2) | 0.006 |
| Preoperative leukocytes (109/L) [mean (SD)] | 6.0 (2.1) | 6.4 (1.9) | 5.8 (2.1) | 0.042 |
| Comorbidities (>1) [n (%)] | 50 (33.8) | 36 (24.3) | 33 (22.3) | 0.059 |
| Diabetes [n(%)] | 13 (8.8) | 11 (7.4) | 14 (9.5) | 0.818 |
| Cardiovascular diseases [n (%)] | 39 (26.4) | 50 (39.9) | 56 (37.8) | 0.031 |
| Pulmonary diseases [n (%)] | 20 (13.5) | 13 (8.8) | 7 (4.7) | 0.030 |
| Kidney failure [n (%)] | 16 (10.8) | 2 (1.4) | 1 (0.7) | <0.001 |
| Neurocognitive disorders [n (%)] | 0 (0) | 2 (1.4) | 1 (0.7) | 0.365 |
| Smoking [n (%)] | 34 (23.0) | 56 (37.8) | 46 (31.1) | 0.021 |
| Previous abdominal surgery [n (%)] | 57 (38.5) | 57 (38.5) | 52 (35.1) | 0.786 |
| Tumour location [n (%)]   - Mid rectum (5 to <10 cm) - Low rectum (<5 cm) | 70 (47.3)  78 (52.7) | 80 (54.1)  68 (45.9) | 79 (53.4)  69 (46.6) | 0.440 |
| Clinical stage of disease (AJCC) [n (%)]   - Stage II - Stage III | 9 (6.1)  139 (93.9) | 56 (37.8)  92 (62.2) | 21 (14.2)  127 (85.8) | <0.001 |

*AJCC, American Joint Committee on Cancer; ASA, American Society of Anaesthesiology; BMI, body mass index and CEA, carcinoembryonic antigen.*

Significant p values are indicated in bold.
